# Opportunities for Ferroptosis in Cancer Therapy

**DOI:** 10.3390/antiox10060986

**Published:** 2021-06-21

**Authors:** Kenji M. Fujihara, Bonnie Z. Zhang, Nicholas J. Clemons

**Affiliations:** 1 Gastrointestinal Cancer Program, Cancer Research Division, Peter MacCallum Cancer Centre, Melbourne, VIC 3000, Australia; bonnie.zhang@unimelb.edu.au; 2 Sir Peter MacCallum Department of Oncology, The University of Melbourne, Parkville, VIC 3010, Australia

**Keywords:** ferroptosis, Eprenetapopt, Erastin, GPX4, glutathione (GSH), SLC7A11, iron, oxidative stress, NRF2

## Abstract

A critical hallmark of cancer cells is their ability to evade programmed apoptotic cell death. Consequently, resistance to anti-cancer therapeutics is a hurdle often observed in the clinic. Ferroptosis, a non-apoptotic form of cell death distinguished by toxic lipid peroxidation and iron accumulation, has garnered substantial attention as an alternative therapeutic strategy to selectively destroy tumours. Although there is a plethora of research outlining the molecular mechanisms of ferroptosis, these findings are yet to be translated into clinical compounds inducing ferroptosis. In this perspective, we elaborate on how ferroptosis can be leveraged in the clinic. We discuss a therapeutic window for compounds inducing ferroptosis, the subset of tumour types that are most sensitive to ferroptosis, conventional therapeutics that induce ferroptosis, and potential strategies for lowering the threshold for ferroptosis.

## 1. Introduction

**Figure 1:**
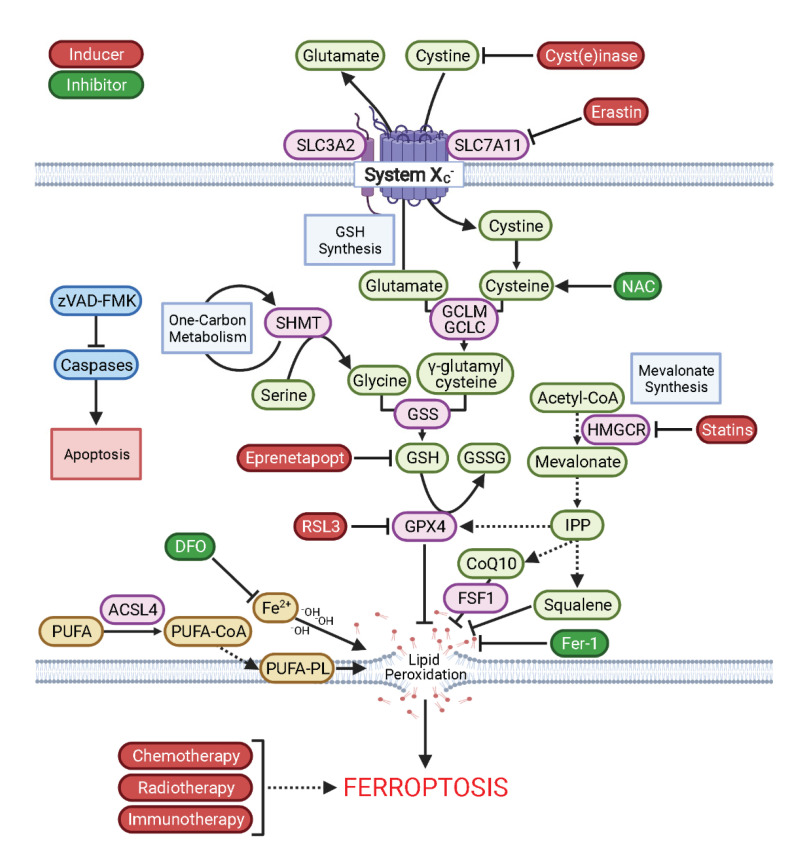
Mechanisms of Ferroptosis Inducers and Inhibitors. Ferroptosis is triggered following the accumulation of iron-catalysed damage to phospholipid-bound polyunsaturated fatty acids (PUFA-PLs). Glutathione peroxidase 4 (GPX4) detoxifies lipid peroxides at the expense of glutathione oxidation (GSH to GSSG). GSH is a tripeptide containing cysteine, glutamate, and glycine and is synthesised through a stepwise pathway catalysed by a glutamate–cysteine ligase catalytic subunit (GCLC), glutamate–cysteine ligase modifier subunit (GCLM), and glutathione synthetase (GSS). Cystine imported in exchange for glutamate by system x_c_^−^ (encoded by *SLC7A11*) provides the main source of cysteine for GSH synthesis. Glycine for GSH synthesis can be sourced from serine catabolism by serine hydroxymethyltransferase (SHMT). Acyl-CoA synthetase long-chain family member 4 (ACSL4) acylates PUFAs, which are incorporated into plasma membranes and are vulnerable to peroxidation. Ferroptosis can be triggered by the GSH depletion (e.g., Cyst(e)inase, Erastin, Eprenetapopt) or direct inhibition of GPX4 (e.g., RSL3). Downstream products of the mevalonate pathway suppress ferroptosis, including isopentenyl pyrophosphate (IPP), which is utilised for selenoprotein synthesis (e.g., GPX4), coenzyme-Q10 (CoQ10) synthesis, which is a co-factor of ferroptosis suppressor protein 1 (FSP1, encoded by *AIFM2*), and squalene synthesis, which is a lipophilic antioxidant. Inhibiting hydroxymethylglutaryl-coenzyme A reductase (HMGCR) with statins amplifies the activity of ferroptosis inducers. Supplementation of exogenous antioxidants (e.g., NAC, N-acetyl-cysteine) to simulate GSH synthesis, lipophilic antioxidants (e.g., Ferrostatin-1, Fer-1) to detoxify lipid peroxides, and iron chelation (e.g., DFO, deferoxamine) blocks the induction of ferroptosis. Pan-caspase inhibitors fail to rescue the cell death (e.g., zVAD-FMK) induced by ferroptosis inducers. Ferroptosis induction by traditional therapies (chemotherapy, radiotherapy, and immunotherapy) contributes to their anti-cancer activity. Figure generated using BioRender.com (21 June 2021).

A hallmark of cancer is the development of resistance to apoptosis, often through genetic loss of the molecular machinery involved in programmed cell death [1]. Furthermore, resistance to chemotherapeutics and molecular targeted therapies are major challenges in oncology [2]. As a result, harnessing our understanding of non-apoptotic cell death pathways, such as ferroptosis, has substantial therapeutic potential for patients, especially in the metastatic setting where effective therapeutic strategies remain limited [3]. Ferroptosis is an iron-dependent, non-apoptotic form of regulated cell death characterised by aberrant lipid membrane peroxidation [4]. As such, the induction of ferroptosis is experimentally verified by the restoration of cell viability by iron chelators and lipophilic antioxidants, and by lack of cell death rescue by pan-caspase inhibitors (Figure 1). Given that dysregulated iron metabolism and iron accumulation have been frequently observed across both solid tumours and haematological malignancies [5], selectively inducing ferroptosis is an attractive potential anti-cancer strategy with broad clinical implications. In this perspective, we discuss the potential for weaponising ferroptosis in the clinic through two therapeutic avenues: (1) triggering ferroptosis in cancer cells directly with targeted agents and (2) lowering the threshold at which cancer cells undergo ferroptosis to enhance the efficacy of conventional therapies, including chemotherapy, radiotherapy, and immunotherapy.

## 2. The Development of Ferroptosis Inducers

Ferroptosis is triggered through two mechanisms, either through the depletion of the cellular antioxidant glutathione (GSH), or through direct inhibition of the enzyme responsible for reversing lipid oxidation, glutathione peroxidase 4 (GPX4). While more detailed reviews of ferroptosis can be found elsewhere [3,6,7], here, we highlight the key compounds used in the elucidation of the mechanisms of ferroptosis. The first chemical agent found to trigger ferroptosis, Erastin, was originally identified in a high-throughput chemical library screen to identify compounds that were selectively lethal in oncogenic mutant HRAS^V12^ cells [8]. Later, the protein target of Erastin was elucidated as system x_c_^−^ (encoded by *SLC7A11* and *SLC3A2*), a cell surface cystine–glutamate antiporter [4,9]. Erastin was found to inhibit the activity of system x_c_^−^, limiting the cellular supply of cystine, which critically leads to the depletion of intracellular GSH. Likewise, cystine deprivation in vitro also induces ferroptosis and phenocopies many of the cell death features induced by Erastin [10]. Furthermore, restricting cystine/cysteine availability to cancer cells through enzymatic degradation with cyst(e)inase triggers ferroptosis and inhibits tumour growth in vivo [11,12]. Moreover, recent work by us and others showed that Eprenetapopt (APR-246, PRIMA-1^MET^), previously identified as a mutant-p53 reactivator, can also induce ferroptosis and has demonstrated capacity to conjugate to free cysteine and deplete GSH [13,14,15].

**Figure 2:**
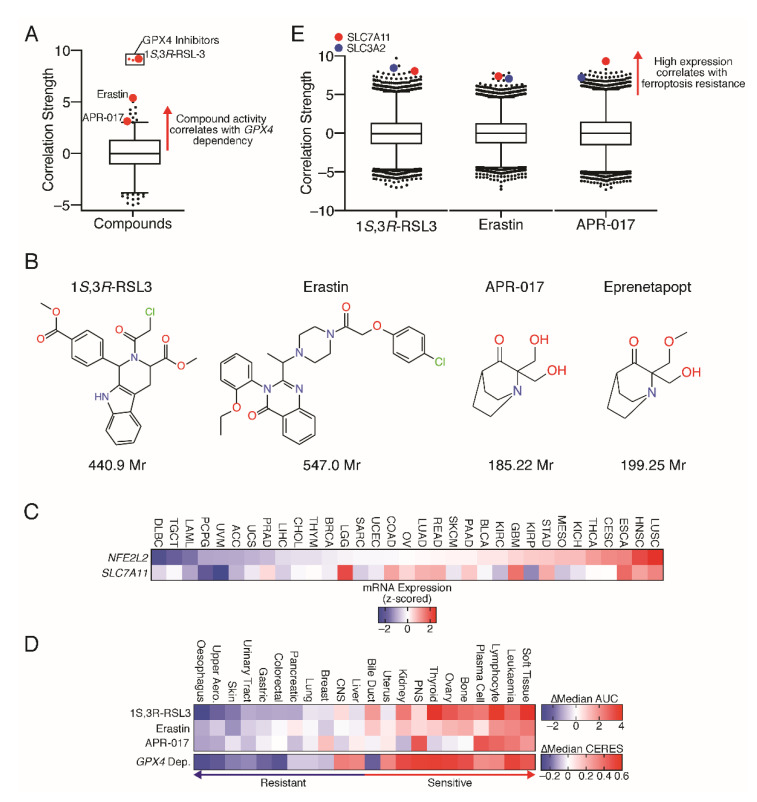
DepMap and TCGA data (**A**) Box-and-whisker plot (1st–99th percentile) of Fischer’s transformed z-scored Pearson correlation strength of *GPX4* dependency and the 481 Cancer Therapeutic Response Portal v2 (CTRPv2) compound activity data across ~700 cancer cell lines. Red dots indicate examples of ferroptosis inducers. (**B**) Chemical structures and molecular weights (Mr) of ferroptosis inducers. (**C**) Heatmap of gene expression of *NFE2L2* and *SLC7A11* in patients with cancer were analysed from the TCGA, cancer type ordered by *NFE2L2* expression. ACC, adrenocortical carcinoma; BLCA, bladder urothelial carcinoma; BRCA, breast invasive carcinoma; CESC, cervical squamous cell carcinoma and endocervical adenocarcinoma; CHOL, cholangiocarcinoma; COAD, colon adenocarcinoma; DLBC, lymphoid neoplasm diffuse large B- cell lymphoma; ESCA, oesophageal carcinoma; GBM, glioblastoma multiforme; HNSC, head and neck squamous cell carcinoma; KICH, kidney chromophobe; KIRC, kidney renal clear cell carcinoma; KIRP, kidney renal papillary cell carcinoma; LAML, acute myeloid leukaemia; LGG, brain lower grade glioma; LIHC, liver hepatocellular carcinoma; LUAD, lung adenocarcinoma; LUSC, lung squamous cell carcinoma; MESO, mesothelioma; OV, ovarian serous cystadenocarcinoma; PAAD, pancreatic adenocarcinoma; PCPG, pheochromocytoma and paraganglioma; PRAD, prostate adenocarcinoma; READ, rectum adenocarcinoma; SARC, sarcoma; SKCM, skin cutaneous melanoma; STAD, stomach adenocarcinoma; TGCT, testicular germ cell tumours; THCA, thyroid carcinoma; THYM, thymoma; TPM, transcripts per million; UCEC, uterine corpus endometrial carcinoma; UCS, uterine carcinosarcoma; UVM, uveal melanoma. (**D**) Heatmap of sensitivity to ferroptosis inducers, RSL3, Erastin, Eprenetapopt analogue (APR-017), and *GPX4* dependency across ~700 cancer cell lines. Cancer lineages ordered by sensitivity. AUC, area under the curve (compound activity); CERES, copy-number adjusted gene dependency score. (**E**) Box-and-whisker plot (1st-99th percentile) of Fischer’s transformed z-scored Pearson correlation strength of RSL3, Erastin and Eprenetapopt analogue (APR-017) activity and genome-wide expression data across ~700 cancer cell lines. Red dot indicates SLC7A11 and blue dot indicates SLC3A2, which encode system x_c_^−^. Data accessed from www.depmap.org (accessed on 30 March 2021) and www.cbioportal.org (accessed on 30 March 2021).

Following on from the discovery of Erastin, 1*S*,3*R*-RSL3 (Ras synthetic lethal-3, RSL3) was identified in an analogous fashion [16]. Here, RSL3, as well as Erastin, were shown to induce cell death through a non-apoptotic, iron-dependent mechanism, and cells transformed with oncogenic RAS had increased levels of iron accumulation due to upregulation of transferrin receptor 1 [16]. Unlike Erastin, however, RSL3 was found to act independently of system x_c_^−^ inhibition [4], and to instead covalently inhibit GPX4 [10], a unique cellular selenoenzyme that reduces phospholipid hydroperoxides to lipid alcohols using GSH as a cofactor [17,18]. As a result, inhibiting GPX4 activity, either directly or indirectly through GSH depletion, triggers unrestricted lipid peroxide accumulation in the presence of iron and subsequently results in the rupture of the plasma membrane [19]. These observations are consistent with our analyses of the Cancer Dependency Map (DepMap) and Cancer Therapeutics Response Portal v2 (CTRPv2) datasets [20,21,22], which highlight that *GPX4* gene dependency correlates with cancer cell line sensitivity to GPX4 inhibitors (including RSL3), Erastin, and APR-017 (analogue of Eprenetapopt) (Figure 2A,B). The DepMap dataset contains gene dependency data generated from pooled genome-wide CRISPR knockout screening of over 1000 cancer cell lines, whilst the CTRPv2 dataset contains compound activity data from 481 compounds across ~700 cancer cell lines. Correlating these datasets can reveal insights into compound mechanisms of action, as demonstrated.

## 3. Therapeutic Index for Ferroptosis

**Figure 3:**
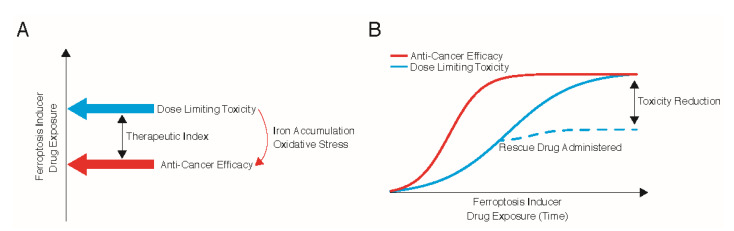
Therapeutic Index for Ferroptosis Inducers. (**A**) Schematic depiction of the proposed therapeutic index for ferroptosis inducers as anti-cancer agents. (**B**) The potential use of rescue drugs following ferroptosis inducer treatment. Rescue drugs could be administered once the maximal efficacy of ferroptosis inducers has been reached to reduce dose limiting toxicities without diminishing the effects on tumours.

All anti-cancer therapeutics principally rely on the selective targeting and destruction of tumour cells over normal cells, known as the therapeutic index. Understanding the differences in the threshold at which cancer cells undergo ferroptosis compared to normal cells is vital for the clinical deployment of ferroptosis inducers, to both mitigate unwanted toxicities and maximise therapeutic benefit [23]. Ferroptosis inducers could be used to leverage the increased levels of oxidative stress and iron in cancer cells to drive their therapeutic index (Figure 3A). Ultimately, the efficacy and safety profiles of ferroptosis inducers can only be established through clinical trials of compounds that have appropriate pharmacodynamics and pharmacokinetics. Whilst Erastin and RSL3 are not readily bioavailable, Erastin analogues (e.g., PRLX 93936), other system x_c_^−^ inhibitors (e.g., Sorafenib, Sulfasalazine), and GPX4 inhibitors (e.g., Altretamine, Withaferin A) are under clinical investigation across various tumour streams [3]. Eprenetapopt is also being tested in a phase III clinical trial in *TP53*-mutated myeloid dysplastic syndromes (NCT03745716). Nevertheless, lessons can be gleaned from the development and usage of cytotoxic chemotherapeutics and targeted therapies in order to achieve the greatest clinical benefit for patients. These include considerations of cancer type and setting, predictive biomarkers, and the use of rescue compounds to mitigate on-target toxicities.

## 4. Oxidative Stress and Iron

Cancer cells experience elevated levels of oxidative stress due to increased reactive oxygen species (ROS) production arising from the augmented metabolic demands to support biomass accumulation and proliferation compared to non-transformed cells [24]. In response, some cancer cells restrict ROS by elevating antioxidant pathways in order to avoid the deleterious effects of oxidative stress [25]. For example, lung cancers frequently harbour mutations in *NFE2L2* (nuclear factor, erythroid 2-like 2, encodes for NRF2) or *KEAP1* (encodes for KEAP1, a negative regulator of NRF2), which results in the activation of antioxidant pathways, including *SLC7A11* and *NQO1* upregulation [26,27]. Furthermore, there is direct evidence supporting the role of NRF2 as a negative regulator of ferroptosis by promoting antioxidant pathways [28,29]. Conversely, NRF2 acts as a guardrail to unchecked cell cycle and proliferation in haematopoietic stem cells [30], and as a result NRF2 and its target genes are found at low levels in haematological malignancies compared to solid tumours (Figure 2C). Iron accumulation is found frequently in several cancer types, especially haematological malignancies [5]. Iron is an important heavy metal required for a multitude of biological processes, including iron–sulphur cluster biogenesis to support mitochondrial metabolism and DNA synthesis, and heme synthesis to support cellular oxygen trafficking. Furthermore, iron participates in ROS generating reactions and lipid peroxidation formation via Fenton chemistry [31]. As a result, the levels of iron accumulation in cancer cells compared to healthy tissues provides a therapeutic index for ferroptosis inducers.

## 5. Cancer Type

As a result of these factors, it is clear that cancer-type specific factors play a role in the sensitivity of tumour cells to ferroptosis, and identifying which tumour types are most likely to benefit will be a key factor in the successful development of ferroptosis inducers as therapeutics. As such, cancer cells of mesenchymal origin were found to be selectively sensitive to ferroptosis inducers compared to epithelial-derived cancer cells [32]. For example, cancers that arise from soft tissue, bone, haematological, and lymphoid tissues (i.e., mesenchymal origin) display high dependency on GPX4 for survival and high sensitivity to ferroptosis activators compared to epithelial-derived cancer cell lines (e.g., oesophageal, upper aerodigestive, and skin; Figure 2D). Further, cancer cells of epithelial origin that have undergone epithelial-to-mesenchymal transition (EMT) are more susceptible to ferroptosis [32]. Increased polyunsaturated fatty acid (PUFA) synthesis in mesenchymal-like state cells likely drives the increased dependency on GPX4 to dissipate reactive lipid peroxides [32,33]. Moreover, breast cancer cells that enter a mesenchymal-like state following Lapatinib treatment become highly sensitive to GPX4 inhibition [29]. Importantly, EMT drives the metastatic potential of cancer cells [34], which suggests that metastatic cells may be more vulnerable to ferroptosis. Cell-to-cell interactions also play a major role in ferroptosis sensitivity; cells plated at a lower density display increased sensitivity to ferroptosis compared to identical but confluent cells in culture [35]. Mechanistically, cell-to-cell contacts rely on E-cadherin, which suppresses ferroptosis through activation of the NF2 and Hippo signalling pathways [35]. This matches the findings relating to EMT and ferroptosis as mesenchymal-like cells lose E-cadherin expression in order to diminish cell-to-cell interactions. Collectively, these findings suggest that cancers of mesenchymal origin, especially haematological cancers, and those prone to EMT and metastasis are likely to be strong candidates for therapeutically leveraging ferroptosis inducers.

## 6. Therapeutic Biomarker

Predictive therapeutic biomarkers could also be utilised to screen and select for patients with a higher probability of response to ferroptosis inducers. A likely beneficial approach would be to screen patient tumours for low SLC7A11 expression, as high expression of SLC7A11 correlates with resistance to ferroptosis inducers (Figure 2E). This further highlights the strong likelihood that patients with haematological malignancies would benefit from treatment with ferroptosis inducers as SLC7A11 expression in haematological cancers is low, correlating with their low NRF2 expression (Figure 2C).

## 7. Use of Rescue Agents to Mitigate Toxicity

Rescue interventions could be used to mitigate the on-target side effects of ferroptosis. For example, chemotherapeutic dosing with methotrexate is often followed by folinic acid supplementation in order to limit haematological and hepatic toxicities [36]. To date, while it is possible to rescue the cell death induced by ferroptosis inducers, no attempts have been made to establish whether the selectivity of ferroptosis inducers for tumour cells could be improved by selectively blocking ferroptosis in normal cells. One could hypothesise that high-dose N-acetyl-cysteine, which is routinely used to treat paracetamol poisoning [37], could be used to rescue normal cells and limit side-effects following treatments with ferroptosis inducers (Figure 3B). Furthermore, iron chelators (e.g., deferoxamine) are commonly used to treat patients with iron overload and could also be utilised to limit deleterious side-effects of ferroptosis inducers.

## 8. Conventional Therapeutics That Induce Ferroptosis

Several recent studies have identified that cancer cells undergo ferroptosis in response to conventional therapeutics, including chemotherapy, radiotherapy, and immunotherapy. Cytotoxic chemotherapies typically target rapidly proliferating cells by interfering with cellular processes involved in cell division and DNA replication. Whilst the evidence indicating that ferroptosis induction directly by conventional chemotherapeutics, such as cisplatin and gemcitabine, is limited [38,39], there is pre-clinical evidence that ferroptosis inducers can synergise with traditional chemotherapeutics [40]. In the case of Eprenetapopt, significant pre-clinical evidence demonstrates the chemosensitisation capacity of ferroptosis activation, including in oesophageal, ovarian, and haematological malignancies [41,42,43,44]. Meanwhile, sorafenib, a multi-tyrosine kinase inhibitor used in the treatment of advanced liver cancer, and sulfasalazine, an anti-inflammatory drug often used to treat rheumatoid arthritis, have both been shown to induce ferroptosis through inhibition of system x_c_^−^ [9,45,46].

Radiotherapy uses high-dose ionising radiation delivered locally to tumour-affected tissues to kill cancer cells, predominantly by causing DNA damage. Radiotherapy is also known to induce oxidative stress in cancer cells by generating reactive oxygen species [47]. Radiotherapy was recently shown to trigger ferroptosis through ATM-mediated suppression of SLC7A11 [48]. Furthermore, upregulation of acyl-CoA synthetase long-chain family member 4 (ACSL4) also contributes to the promotion of ferroptosis by radiotherapy [49]. ACLS4 preferentially acylates long-chain PUFAs [50], which are incorporated into plasma membranes, increasing the membrane-resident pool of oxidation-sensitive lipids [51]. Interestingly, whilst ferroptosis inhibitors partially block the cell death induced by radiotherapy, they do not block the DNA damage triggered by ionising radiation [49]. Importantly, strong synergy was reported between radiotherapy and ferroptosis activators, including cyst(e)inase and sulfasalazine [48,49].

Immunotherapy with immune checkpoint inhibitors (ICI) has revolutionised clinical care of cancer patients, providing an additional pillar to the suite of cancer treatment modalities. ICI predominantly elicit their anti-cancer effects by inhibiting tumour cell capacity to dampen cytotoxic T cell-mediated killing. ICI have also been shown to induce CD8^+^ T cell-mediated ferroptosis through suppression of SLC7A11 and SLC3A2 by interferon gamma (IFNγ) released by the T-cells [52]. Further, PD-L1 blockage therapy synergised with ferroptosis inducers, Erastin, RSL-3, and cyst(e)ine, both in vitro and in vivo [52], as well as with radiotherapy [48]. More recently, high expression of the receptor tyrosine kinase, TYRO3, was shown to correlate with resistance to anti-PD-1 therapy and suppress the induction of ferroptosis in tumour cells by activating NRF2 [53]. Conversely, ferroptosis inducers have also been shown to induce ferroptosis in CD8^+^ T cells, limiting the anti-cancer efficacy of ICI [54]. Preliminary results also reported that Eprenetapopt and anti-PD-1 therapy synergise in murine solid tumour models [55]. This has prompted the initiation of a phase I clinical trial to test the safety of Eprenetapopt and anti-PD-1 therapy, Pembrolizumab, in solid tumour malignancies (NCT04383938). Given the tension between how ferroptosis inducers affect ICI and T cell killing, trials like this will provide pivotal insights into the role of ferroptosis inducers as adjuvants to ICI.

It is likely that a portion of the cell death induced by most conventional therapeutic regimens is ferroptotic. However, to date, there has been no systematic attempt to quantify the contribution of cell death driven by ferroptosis in an in vivo or clinical setting with any therapeutic regimen. Such a study could potentially provide rationale for when and how ferroptosis inhibitors or sensitisers should be applied in combination with conventional chemotherapies to maximise therapeutic gain, particularly in the setting of tumour resistance to apoptosis.

## 9. Lowering the Threshold for Ferroptosis

Given that ferroptotic cell death is in part responsible for the tumour killing achieved by chemotherapy, radiotherapy, and immunotherapy, reducing cancer cell capacity to evade ferroptotic cell death would be a powerful therapeutic strategy. Following on from the study investigating ferroptosis sensitivity and mesenchymal-like cell state, statins, common anti-cholesterol drugs, were also identified as modulators of ferroptosis sensitivity in mesenchymal cells [32]. Statins inhibit the rate-limiting enzyme in the mevalonate synthesis pathway, HMG-CoA reductase (HMGCR), which decreases cholesterol abundance (Figure 1). Previous reports detailed that statins inhibit isopentenylation of the selenocysteine-charged transfer RNA, which is required for synthesis of selenoproteins like GPX4 [56]. In keeping with this, statins synergise with GPX4 inhibitors through decreasing the abundance of the GPX4 protein and inducing lipid peroxidation [32]. The mevalonate synthesis pathway also directs several other downstream pathways involved in ferroptosis, including CoQ10 and squalene synthesis—GSH-independent mechanisms of protection against ferroptosis (Figure 1) [57,58,59]. As a result, targeting the mevalonate synthesis pathway with statins could be utilised to lower the threshold at which cancer cells undergo ferroptosis.

Limiting endogenous supply of other key nutrients may provide an alternative strategy to sensitise tumours to undergoing ferroptosis. Chronic activation of the antioxidant response induced by NRF2 activation increases the demand for the supply of glutamine and other non-essential amino acids, due to the increased efflux of glutamate from SLC7A11 to supply cyst(e)ine for GSH synthesis [60,61]. In addition, we recently demonstrated that limiting the availability of serine and glycine (SG) through dietary restriction significantly enhanced the efficacy of Eprenetapopt in vivo by limiting the availability of glycine required for de novo GSH synthesis [13]. However, this differs from the effects of ferroptosis induction by Erastin under SG restricted conditions seen in other studies, where Erastin treatment was found to reverse the sensitivity of *KEAP1* mutant tumour limits to SG restriction [60]. This is likely explained by how Eprenetapopt and Erastin differ in their mechanisms of GSH depletion and effects on cyst(e)ine and glutamate availability (Table 1). Recently, arginine deprivation also demonstrated protection against Erastin and cystine depletion but not against GPX4 inhibition with RSL3 [62]. Given that cyst(e)ine are considered non-essential amino acids, dietary restriction of cystine could provide improved therapeutic benefit for Erastin or other SLC7A11 inhibitors in vivo.

## 10. Conclusions

The identification of ferroptosis as a non-redundant, regulated cell death pathway opens up opportunities for circumventing tumour cell resistance to other forms of regulated cell death, such as apoptosis. Exploiting ferroptosis in cancer therapy requires continued building of our understanding of the mechanisms underlying this cell death pathway; in particular, identification of molecular regulators of sensitivity and resistance to ferroptosis will be crucial for future clinical application. Complementary to this will be the identification of certain tumour types or cell states (such as a mesenchymal phenotype) that are particularly amenable to induction of ferroptosis. Whilst there is active interest in the development of specific ferroptosis inducers as novel therapeutics, the recognition that ferroptosis can be engaged by many current treatment modalities opens up the potential for strategies to leverage this activity indirectly by lowering the threshold for activation of ferroptotic cell death. Given the dependence on specific metabolic pathways to protect tumour cells from ferroptosis, the application of specific diets concurrent with anti-cancer treatments holds much interest and future potential.

## Figures and Tables

**Table 1:** Effect of ferroptosis inducers of GSH, cyst(e)ine uptake, glutamate release, and serine/glycine (SG) restriction.

Inhibitor	GSH Depletion	Cyst(e)ine Uptake	Glutamate Release	SG Restriction
Eprenetapopt	Yes	Increases	Increases	Increases activity
Erastin	Yes	Decreases	Decreases	Decreases activity
1S,3R-RSL3	No	Unknown	Unknown	Unknown

## Data Availability

Data used in Figure 2 is publically accessible at www.depmap.org and www.cbioportal.org.
